# A Novel Well Drill Assisted with High-Frequency Vibration Using the Bending Mode

**DOI:** 10.3390/s18041167

**Published:** 2018-04-11

**Authors:** Xinda Qi, Weishan Chen, Yingxiang Liu, Xintian Tang, Shengjun Shi

**Affiliations:** State Key Laboratory of Robotics and System, Harbin Institute of Technology, Harbin 150001, China; 7120810204@hit.edu.cn (X.Q.); cws@hit.edu.cn (W.C.); tangxintian@hit.edu.cn (X.T.); sirssj@hit.edu.cn (S.S.)

**Keywords:** well drilling, superimposed vibrations, bending vibration, piezoelectric transducer

## Abstract

It is important for companies to increase the efficiency of drilling as well as prolong the lifetime of the drilling tool. Since some previous investigations indicated that a superposition of well drilling with an additional vibration increases the drilling efficiency, this paper introduces a novel well drill which is assisted with additional vibrations by means of piezoelectric sandwich bending vibration transducer. The proposed drill uses bending vibrations in two different directions to from an elliptical trajectory movement, which can help the drill to break the surface of hard material more efficiently and clean away the lithic fragments more easily. The proposed well drill with bending vibration transducer is designed to have a resonance frequency of the first bending vibration mode of about 1779 Hz. The motion equation of the particle on the edge of the drill bit is developed and analyzed. The vibration trajectory of the particle on the edge of the drill bit is calculated by using finite element method. A prototype of the proposed drill using bending vibrations is fabricated and tested to verify the aim of drilling efficiency increase. The feed speed of the vibration assisted drilling is tested to be about 0.296 mm/s when the excitation voltage of the transducer is 300 V, while this speed decreases to about 0.195 mm/s when no vibration is added. This comparison shows that the feed speed of the vibration assisted drilling is about 52% higher than that of the normal drilling, which means the proposed drill has a better efficiency and it is important to consider vibration superimposition in well drilling. In addition, the surface of the drill hole gained by the vibration assisted drilling is smoother than that of the normal drilling, which makes the clearance easier.

## 1. Introduction

Geological mining usually needs to drill deep holes into the ground to detect the condition underground and find out if there are some valuable mineral resources. With the increasing demand of geology prospection, drill holes need to be deeper to find more information about the rock stratums. The rock stratums are usually harder to break as the drilling process becomes deeper, which decreases the efficiency of the drilling. The cost of the deep drilling is much higher than the normal well drilling and the ways of increasing the efficiency of drilling are demanded imminently. 

The percussive-rotary drilling is a popular method in well drilling to improve the drilling conditions and increase the efficiency [1,2,3,4,5]. The percussive-rotary drilling is a drilling method which contains normal rotary drilling movement and axial percussion to help break the surface of rock. The percussive-rotary drilling can be more efficient than the normal method, especially in the conditions where the rocks are hard, such as marble, shale and so on. In this method, the drill bit collides with the rock with high intensity, which can help the drilling flush out rock fragments [6]. There are some important advantages lying in the percussive-rotary drilling. Firstly, it is more efficient than the normal rotary drilling in the same condition (such as the same pressure and the same material). Secondly, the lifetime of the drilling bit is longer because the actual collision time between the rock and the bit only takes a small percentage of the total drilling time, while it takes more percentages in normal drilling method. Thirdly, the hole deviation angle is smaller in the percussion method than the normal method. 

The percussive-rotary drilling is mainly accomplished by a device called hydraulic impactor [7,8]. This device uses springs and pistons to control the pressure of drilling fluid to make the fluid impact a cavity in it to generate the axial percussion. However, the hydraulic impactor and other mechanical impactors have some limitations. Firstly, their volumes are large and it is difficult for them to be optimized to have the best percussion effects and amplitudes. Furthermore, although these devices can increase the efficiency of drilling, they can only produce axial percussion. Therefore, other methods of percussion are waiting to be proposed and tested.

Since the piezoelectric actuator is relatively small and convenient to manipulate, high frequency percussion can be generated on the drill by using a piezoelectric bending transducer [9,10,11,12,13,14,15]. There have been studies which showed that the ultrasonic assisted drilling has been used in brittle ceramic material drilling, such as micro-hole drilling of glass and extraterrestrial rock drilling [16,17,18]. However, those studies only used the longitude vibrations along the axis of the drill but did not consider other vibration modes. Therefore, a drill with bending vibrations is designed through this idea, which has possibilities to be more efficient than the normal rotary drilling. Altogether, the following advantages can be anticipated: better surface condition, higher drilling efficiency and smaller chips (easier for removal).

## 2. Drill Structure and Transducer Design

To superimpose a vibration on the drill bit, the vibration actuator should be placed near the drill bit to transmit most of the energy to the end tip of the drill. The actuator should be placed in the drill stem and near the bit rather than in the drill bit itself because it is relatively difficult to redesign the drill bit. The vibration actuator uses piezoelectric material rather than magnetostrictive material as the excitation element because of the practical demand of preventing strong magnetic fields in well drilling. 

**Figure 1:**
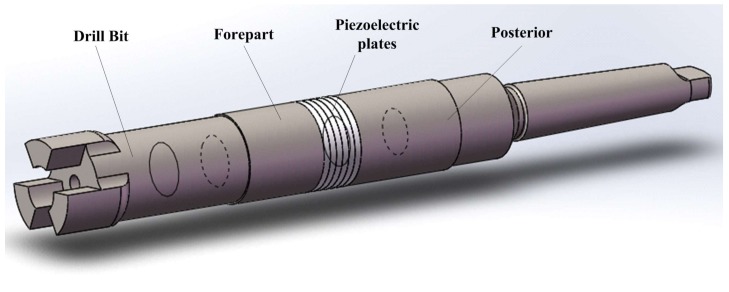
The structure of the proposed well drill with bending vibration transducer.

**Figure 2:**
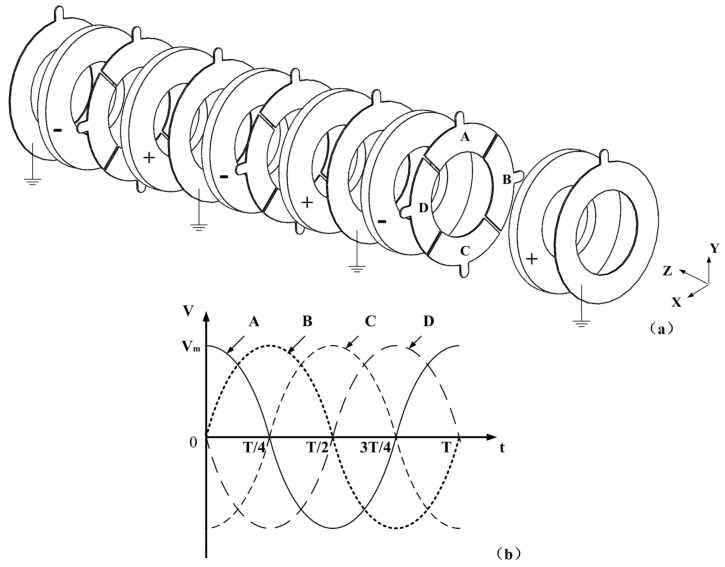
The arrangements of the piezoelectric ceramic plates, the electrodes and the excitation voltages: (**a**) the arrangements; and (**b**) the excitation signals.

For the drill structure, it can be divided into four parts: drill bit, forepart of the drill, piezoelectric transducer and posterior of the drill (Figure 1). There is a structure of standard cone rod with Morse taper number 4 at the tail of the posterior, which is used to connect the drill with bench drill as well as transmit rotary movement. For the transducer, the piezoelectric ceramic plates should be placed at the antinode of the bending vibration mode. The arrangements of the piezoelectric ceramic plates and the electrodes are shown in Figure 2, in which “+” and “−” are used to illustrate the polarizations of the piezoelectric ceramic plates.

The electrodes are placed between the piezoelectric plates to apply the excitation signals. In each piezoelectric plate, the direction of polarization is the same. Some electrode slices are cut apart into four independent equal pieces, on which four different excitation signals, A, B, C and D, are applied. In detail, the two excitation signals of A and C have a phase difference of 180°, and they can generate a bending vibration around X axis. The other bending vibration around Y axis is produced by the two excitation signals of B and D. Therefore, two bending vibrations can be generated by a single group of piezoelectric ceramic plates by applying four different excitation signals. Six piezoelectric plates are used to enhance the power of the vibration as the drill structure is relatively huge. The bending vibrations of the two orthogonal directions can be controlled by the excitation signals. When these two bending vibrations have the same frequency and a phase difference of 90°, they can be coupled together and the movement on the drill will have a constant trajectory. 

To design an efficient and practical drill, several kinds of parameters should be determined, which include fix parameters, vibration related parameters and variable parameters. The fixed parameters contain the external diagram of the drill stem, the feed speed of the drill and so on, which are mainly related to the drilling process. These parameters are determined from existing information.

**Figure 3:**
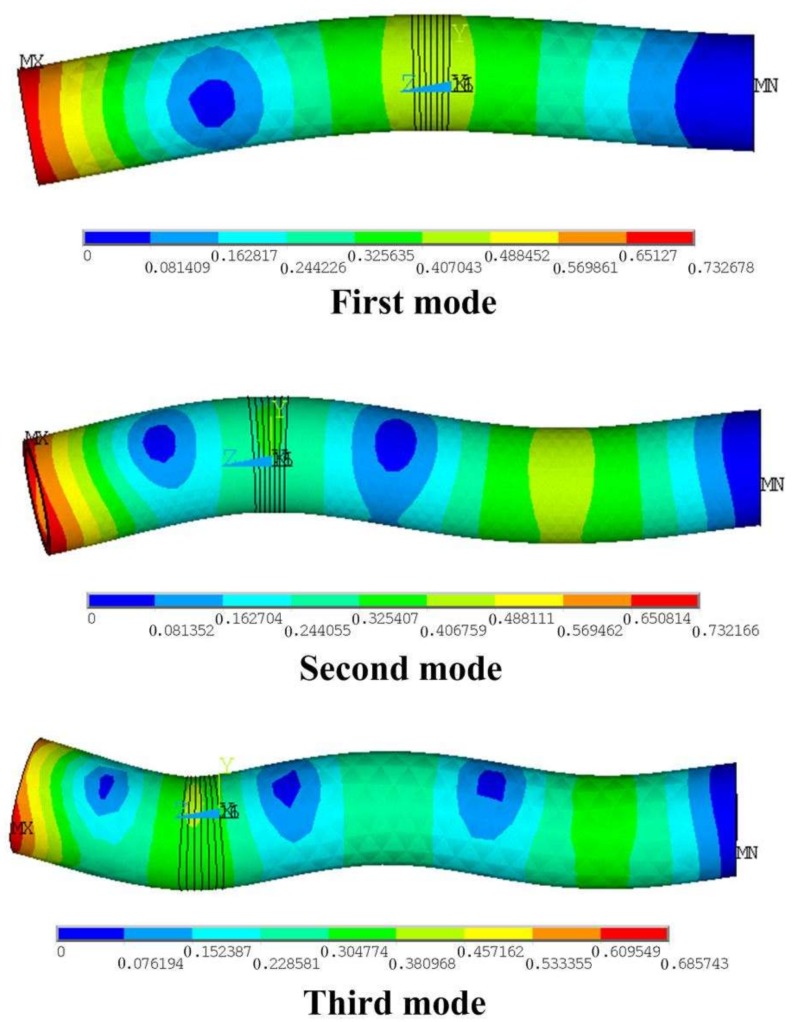
The low order vibration modes of the transducer.

**Figure 4:**
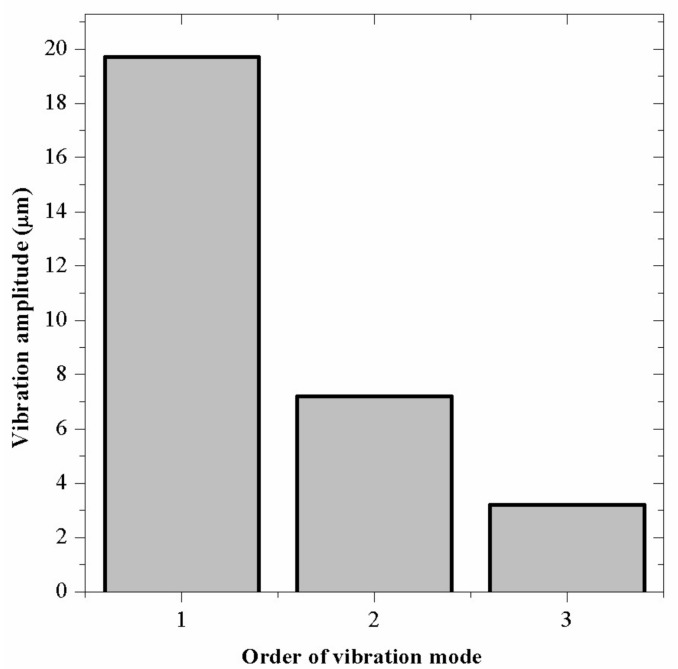
The vibration amplitudes under different modes.

The vibration related parameters include the resonant frequency and the vibration amplitude. Since relatively large amplitude of vibration is required to make the efficiency improvement remarkable, bending vibration mode of relatively low order is needed. Three basic order vibration modes can be considered: the first, the second and the third bending mode, respectively, as shown in Figure 3. To find a proper vibration mode, differences between these modes are calculated by finite element method (FEM, ANSYS Software, version 10.0). Three simplified drills are used in these primitive calculations, which only have drill stems and the piezoelectric ceramic plates. The vibration amplitudes of these modes under the same excitation voltage of 300 V are shown in Figure 4. The vibration amplitude is not the only factor to decide the vibration energy: the vibration frequency is also significant. The vibration amplitude is selected as the main parameter to design the transducer in this work. From the results, the first mode is selected for the design and the experiment because large vibration amplitude is required.

**Figure 5:**
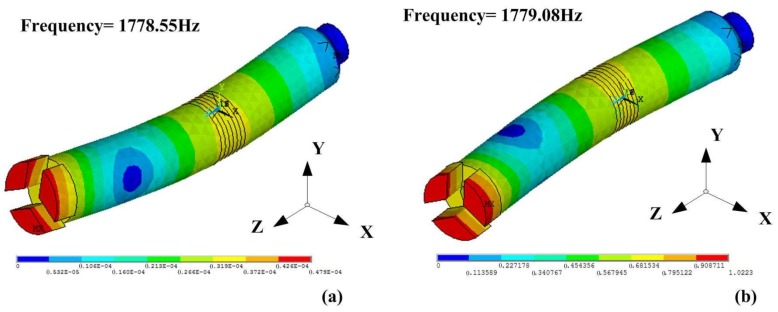
The modes of the bending vibrations in two directions: (**a**) the vibration mode in Y direction; and (**b**) the vibration mode in X direction.

After the preliminary analysis, the two bending vibrations should be coupled so the drill bit has a constant vibration trajectory during the drilling process, which means that they should have the same resonance frequency and a constant phase difference. The resonant frequencies of the two bending modes in X and Y direction are nearly the same as the structure of the drill is almost centrosymmetric. The FEM transient analysis shows that their resonance frequencies are about 1779 Hz, as shown in Figure 5, which makes the mode coupling become easy. Thus, when four excitation signals with the same frequency and the same amplitude, but different initial phases of 0°, 90°, 180° and 270°, respectively, are applied on the electrodes, and the frequency is the same with the resonant frequency of the bending modes, an efficient and constant vibration will be generated on the drill. 

In addition, since each piezoelectric plate can generate two bending vibrations by itself, no matter where the piezoelectric actuator in the drill is, the amplitudes of the two vibrations will be the same when signals with the same amplitude are applied. Thus, the design of the piezoelectric plates in the transducer improves the symmetry of the drill and makes the mode coupling become easier. 

The variable parameters should be designed to determine the size and shape of the drill and the location of the piezoelectric plates. In addition, they should be designed to amplify the vibration. The location of the piezoelectric plates in the drill is gradually moved to the antinode by using modal analysis for the aim of high efficiency. 

**Figure 6:**
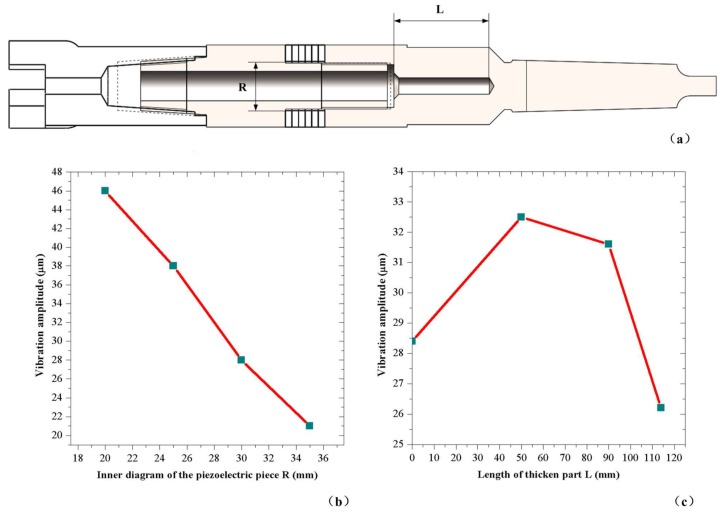
The analysis of the structure parameters of the drill: (**a**) the drill and its parameters; (**b**) influence of the inner diagram of the piezoelectric plate on the vibration amplitude; and (**c**) influence of the length of the thickened part on the vibration amplitude.

Other variable parameters of the drill can be determined by the FEM transient analysis to generate a more intense vibration at the drill bit under the limits of other parameters. First, appropriate radial sizes of the transducer and the drill should be confirmed to let the vibration become violent. By applying the same excitation voltage and changing the main radial parameter, the vibration amplitudes are calculated, as shown in Figure 6b. There is a tendency that the vibration amplitude increases when the inner diameter of the piezoelectric plate decreases. However, the diameter of the drill’s inner hole cannot be too small due to the limit of the screw. In this condition, the relatively best radial parameters are determined from the structure limit. 

Next, to amplify the vibration amplitude, the back of the drill should be thickened. The length of the thickened part is associated with the shape of vibration mode and the enhancement effect. A result is calculated and shown in Figure 6c by using FEM analysis and changing the length of the thicken part. In the calculations, the same excitation voltage is applied and the locations of piezoelectric plates have been adjusted to the antinode of the bending vibration. An appropriate length of the thicken part is determined to be 50 mm to get the best amplifying effect.

## 3. Motion Analysis

During the vibration assisted drilling process, the drilling bit rotates and vibrates in hybrid bending modes. The speed of the rotary motion is set as 70 r/min, which is suitable for a smooth drilling; it can prevent the excessive wear of the drill bit and is safe for researchers to conduct experiments. In addition, rotary speed of 70 r/min is a reachable speed of the bench drill used in the experiments. The hybrid bending vibrations drive the drill bit to make a high-speed movement because of the frequency (1779 Hz).

In the analysis, sinusoidal AC voltage is the input signal of the transducer. Since the hybrid bending vibration modes are made of two independent bending modes of different directions, a simple harmonic vibration will be generated at the drill bit in the same direction if sinusoidal AC voltage is applied on the transducer in one direction. A particle P on the edge of the drill bit is chosen to be analyzed.

In X direction, the vibration movement can be described as:

(1) $$X_{p}=A_{X}\cos\left(2\pi f_{A}t+\alpha \right)$$

in which *X_p_* is the displacement of particle P in X direction; *A_X_* is the amplitude of the vibration in X direction; *f_A_* is the frequency of the bending vibration in X direction; *t* is the time; and *α* is the initial phase of the vibration in X direction.

In addition, in Y direction, the movement can be described as:

(2) $$Y_{p}=A_{Y}\cos\left(2\pi f_{B}t+\beta \right)$$

in which *Y_P_* is the displacement of particle P in Y direction; *A_Y_* is the amplitude of the vibration in Y direction; *f_B_* is the frequency of the bending vibration in Y direction; and *β* is the initial phase of the vibration in Y direction.

In the analysis, the two excitation voltages have the same frequency and the difference between *α* and *β* is 90°. Additionally, the amplitudes of the voltages are equal too. Thus, the movement of the drill in XOY plane can be described as:

(3) $$X_{p}^{2}+Y_{p}^{2}=A^{2}$$

in which *A* is the radius of the trajectory of particle P. 

Thus, the vibration trajectory movement is a circle in this condition. Using the frequency and the amplitude of the bending vibration, the speed of the vibration movement can be calculated from:

(4) $$v_{1}=2\pi fA$$

in which *ν*_1_ is the vibration speed of particle P.

The rotary speed of the drill can be calculated from:

(5) $$v_{2}=2\pi nR$$

in which *ν*_2_ is the rotary speed of particle P; *n* is the rotate speed of the bench drill; and *R* is the distance between particle P and the axis of the drill.

When the drill has both rotation and vibration, the displacement and velocity equations of the particle P on the drill’s cutting edge are deduced as:

(6) $$\left\{ \begin{array}{l} X=R\cdot \cos\left(2\pi n\cdot t+\varphi_{1}\right)+A\cdot \cos\left(2\pi f\cdot t+\varphi_{2}\right)\\ Y=R\cdot \sin\left(2\pi n\cdot t+\varphi_{1}\right)+A\cdot \sin\left(2\pi f\cdot t+\varphi_{2}\right)\\ v_{x}=2\pi nR\cdot \sin\left(2\pi n\cdot t+\varphi_{1}\right)+2\pi fA\cdot \sin\left(2\pi f\cdot t+\varphi_{2}\right)\\ v_{y}=2\pi nR\cdot \cos\left(2\pi n\cdot t+\varphi_{1}\right)+2\pi fA\cdot \cos\left(2\pi f\cdot t+\varphi_{2}\right) \end{array}\right.$$

in which *X* is the displacement of particle P in X direction; Y is the displacement of particle P in Y direction; *ν _x_* is the velocity of particle P in X direction; *ν_y_* is the velocity of particle P in Y direction; *t* is the time; *φ*_1_ is the initial phase of the rotation; and *φ*_2_ is the initial phase of the vibration.

When the direction of rotary speed of the drill is used as a reference direction, the velocity and the acceleration equations of the particle P in the reference direction are deduced as:

(7) $$\left\{ \begin{array}{l} v_{ref}=2\pi nR+2\pi fA\sin\left(2\pi f\cdot t+\varphi_{3}\right)\\ a_{ref}=4\pi^{2}f^{2}\cos(2\pi f\cdot t+\varphi_{3}) \end{array}\right.$$

in which *ν_ref_* is the velocity of particle P in the reference direction; *a_ref_* is the acceleration of particle P in the reference direction; and *φ_3_* is the initial phase of the vibration in the reference system.

**Figure 7:**
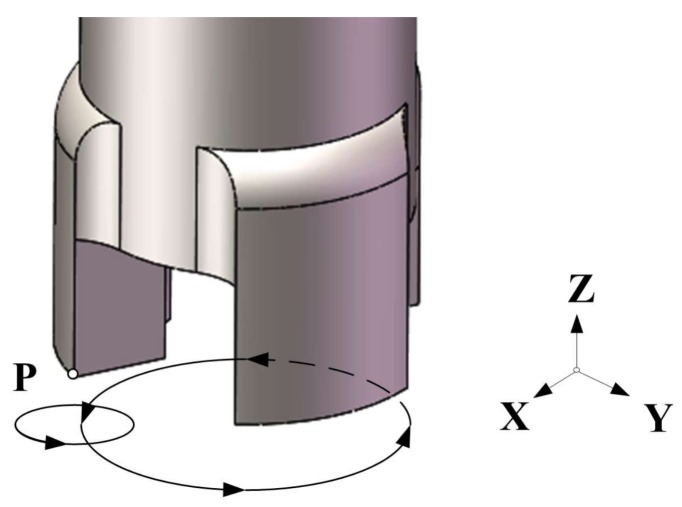
The compound motion of particle P.

**Figure 8:**
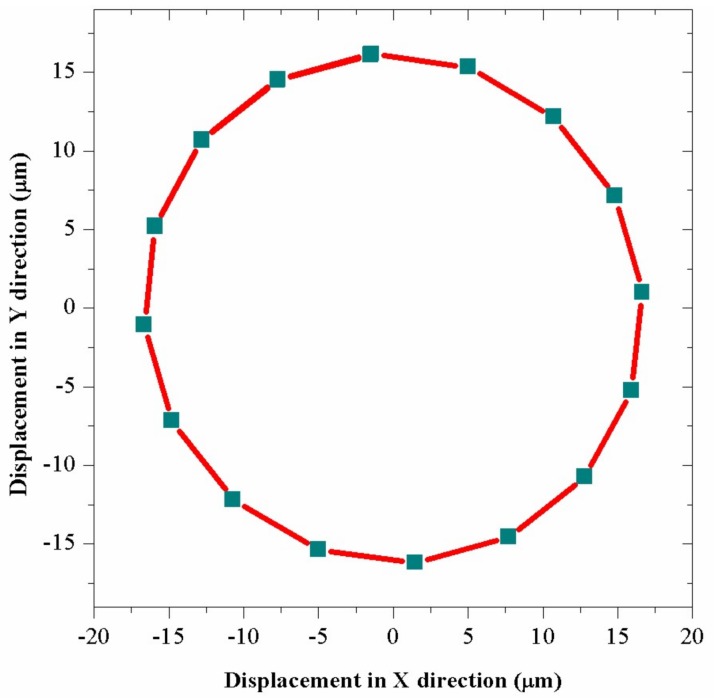
The vibration trajectory of particle P in XOY plane.

During the drilling, there are two basic rotary movements of the drill bit in XOY plane, which are shown in Figure 7, and the movement of particle P is similar to satellite movement in the reference frame of a star. The cutting edge of the drill not only rotates around the axis of the drill, but also has a rotary vibration motion in a small scale. Since the resonant frequency of bending transducer is 1779 Hz, the vibration trajectory of particle P can be calculated and shown in Figure 8 by applying excitation voltage with parameters of 1779 Hz and 200 V in the FEM transient analysis. The result shows that the edge of the drill bit has a circular trajectory vibration in XOY plane, whose amplitude is about 16.2 μm and the vibration speed is calculated to be about 181 mm/s.

The speed of the rotary motion is calculated to be 183 mm/s by Equation (5). The vibration is strong enough to have a great influence on the drilling process because the vibration speed is nearly the same with the speed of rotary motion. In addition, Equation (7) shows that the vibration makes the drill have an additional and sinusoidal acceleration in the direction towards the surface of the drilling material, which can help the drill edge impact the surfaces of the brittle material repeatedly and break them. During the actual drilling process, the edge of the cutting part of the drill bit vibrates quickly while it scrapes and beats the brick material repeatedly. Therefore, the material in the surface layer will be broken up before the rotary motion clears the chips and makes the drill bit go downward, which can make the drilling become more efficient. However, the drill bit relies on the sharpness of the drill edge and the huge pressure on the drill to break the material and then clears the fragments away in the normal rotary drilling; the power of rotary motion is partly used to break the surface of the material, so the drill needs more power and higher pressure to reach the same feed speed of the vibration assisted drilling, which causes that the normal rotary drilling is less efficient and the wear of the drill bit is greater.

**Figure 9:**
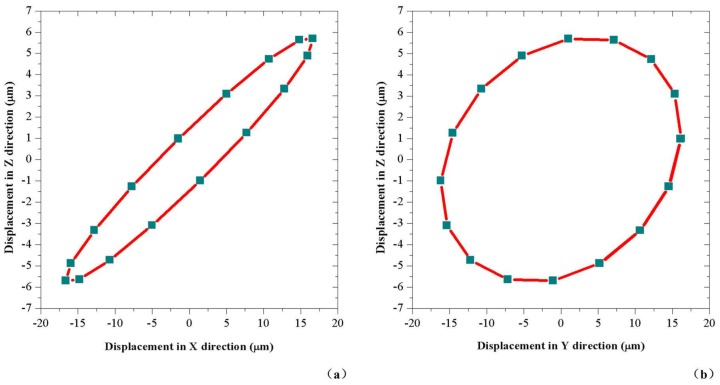
The vibration trajectories of particle P: (**a**) in XOZ plane; and (**b**) in YOZ plane.

In addition, the vibration movement of the cutting edge on the drill bit in other different directions should also be considered because the cutting edge is the main part of the drill to cut materials. The vibration trajectories of the particle P in XOZ and YOZ plane are calculated and shown in Figure 9, by using the FEM transient analysis and applying excitation voltages whose parameters are 1779 Hz and 200 V. In these figures, it is concluded that the particle P on the edge of the drill bit has a vibration in Z direction with amplitude of 5.7 μm during the vibration; because particle P is not located on the axis of the drill, the bending mode can also cause axial displacement. The two movements in XOZ plane and YOZ plane can also help the drill edge scrape the surface of the material and beat the material downwards frequently, which makes the drilling process become more efficient. Moreover, the motions in these two planes are not simple axial percussions but hybrid percussions, which can not only break up the surface of the material but also help to clean the rock fragments.

## 4. Experimental Environment Design

**Figure 10:**
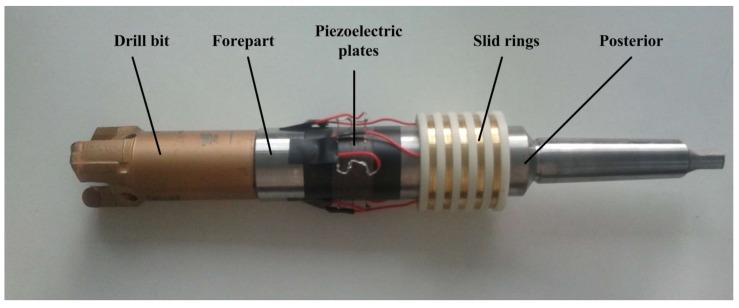
The prototype of the experimental drill.

From the design of the drill and the transducer, an experimental drill was fabricated by several materials. The materials used are listed in Table 1 and the drill is shown in Figure 10.

**Figure 11:**
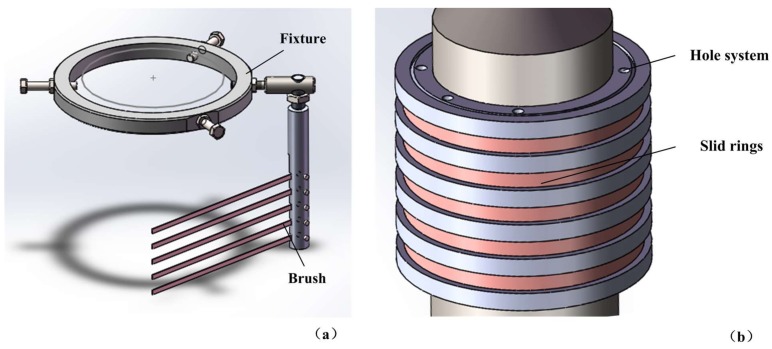
The electric brush: (**a**) electric brush and its fixture; and (**b**) slid rings.

Since the drill was connected to a bench drill to rotate and feed downward, a simple brush device was designed, which was used to conduct electric signals to the transducer and prevent the coiling of the wires. The fixture of the brush was made of steel, as shown in Figure 11a. The slip rings are shown in Figure 11b. The fixture of the brush was clamped with a part of the bench drill which fed downward together with the drill but did not rotate. Thus, the brush and the slip ring became relatively still and could contact with each other during the drilling process.

The connections of the fixture and the brush were screws and there was a fasten bolt on each joint. The fasten bolts ensured the brush and fixture connected tightly during the violent vibration conditions. The brush could rotate a small angle to let the bronze bars cling on the corresponding slid rings with some pressure because the bronze bars on the brush were quite elastic. After this operation, the fasten bolts were used to fix the brush, which helped the electric brush operate reliably.

The slip rings were placed on the drill stem and linked to the electrodes that helped to excite the piezoelectric plates. The rings and the brush were made of bronze and their substrate material was polyamide which had good insulation. There were several hole systems in the substrate structure of the slip rings, which were used to extract the wires attached to the bronze rings and connect them to the electrodes.

The drilling material was the common fireclay brick, which was suitable for drilling because its moderate hardness allowed the experiment to be conducted smoothly and prevented excessive wear of the drill bit. To control variable, several bricks with the same humidity and the same material were used in the experiments. 

**Figure 12:**
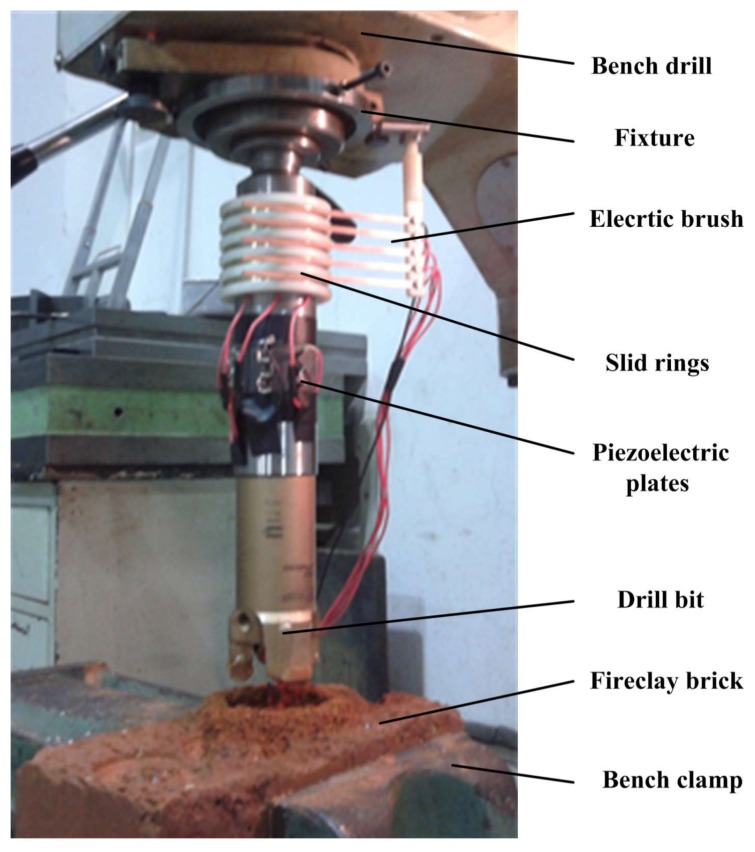
The experimental setup.

The experiment was based on a bench drill which could connect the drill through a cone bar. The experimental setup is shown in Figure 12. An electric brush was connected to the bench drill and the slip rings were placed on the drill stem, which could prevent coiling of wires. During the experiment, the rotary motor of the drill bench was used to provide the power for the rotation of the drill directly. Several loads were suspended around the hand wheel of the bench drill, which provided a constant torque to drive the machine and gave a constant force to feed downward. The precise value of the drilling force was measured by a force meter. The piezoelectric transducer was connected to an AC power source which could control the vibration condition of the drill to conduct comparison experiments. Since the fireclay brick had a regular shape, it was clamped by the clamper of the bench.

## 5. Drilling Experiment Results and Analysis

**Figure 13:**
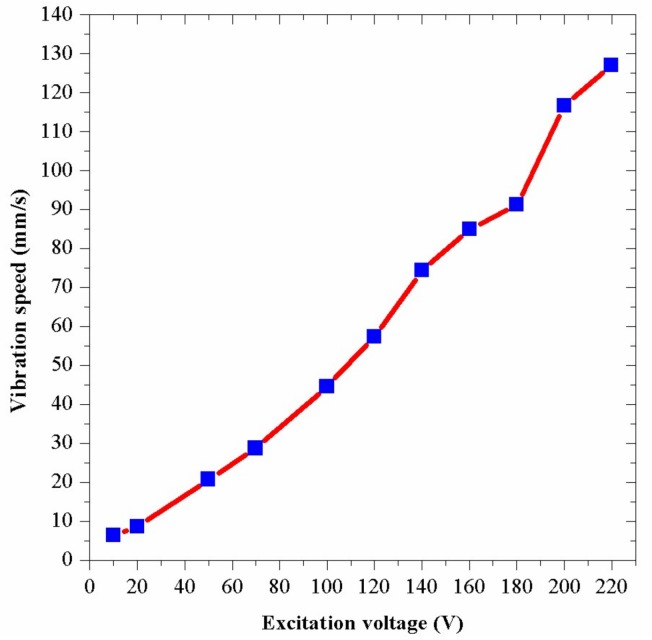
The vibration speeds under different excitation voltages.

After the assembling of the drill, an experiment of the relationship between the vibration speed and the excitation voltage was carried out in the condition of no-load, which could test the actual vibration condition of the drill. Firstly, the resonant frequency of the bending transducer was measured to be 1269 Hz by a laser scanning vibrometer. Next, by applying the excitation voltages of 1269 Hz, the drill began to vibrate efficiently and the vibration speeds were measured by the laser scanning vibrometer. Figure 13 shows the result of the experiment. The experimental curve shows that the vibration speed increases almost proportionately with the increase of the excitation voltage. In this figure, the vibration speed is about 117 mm/s when the excitation voltage is 200 V. By using Equation (4), the vibration amplitude is calculated to be 14.7 μm. Nevertheless, the above FEM analysis has shown that the vibration amplitude is about 16.2 μm when the excitation voltage is 200 V.

The actual vibration amplitude of 14.7 μm is lower than the theoretical analysis result of 16.2 μm. In addition, the actual resonant frequency of 1269 Hz is lower than the theoretical analysis result of 1779 Hz. These discrepancies are caused by several reasons. Firstly, materials of the drill are not ideal and have many flaws, especially in the piezoelectric ceramic plates. Secondly, there are a great quantity of screws and gaps in the drill structure, which can decrease the vibration energy. However, in the theoretical analysis, the joints of the parts are ideal and there is no crack between them. Thirdly, since the real structure of the drill bit is very complex, the model of the drill has been simplified to carry out theoretical analysis quickly, which also affects the frequency and the vibration amplitude of the analysis. Fourthly, the theoretical analysis result is achieved under a fix boundary condition of the posterior; however, this part is linked with the bench drill in the experiments, which changes the boundary condition. In the experiments, the boundary condition change can be viewed as an increasing of the length of the bending transducer, which can result in the decrease of the resonance frequency and the vibration amplitude.

For the test of the efficiency of the vibration assisted drilling, some actual drilling experiments were conducted, which used a constant drilling force and took the feed speeds of the drilling as the results to show the efficiency of the drilling. The speeds were calculated by the period times in which the drill fed 5 mm downward in the bricks. The efficiencies of these test groups were compared by experiments using drills superimposed with different vibrations and no vibration.

**Figure 14:**
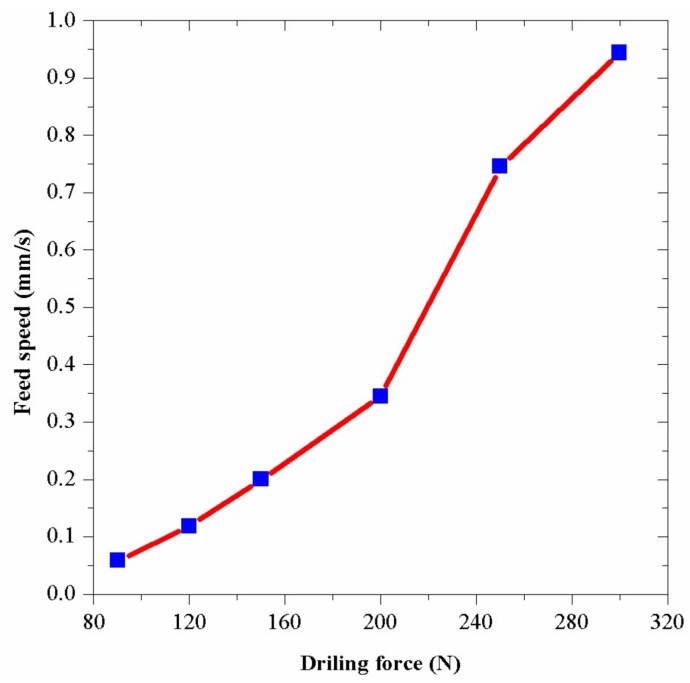
The feed speeds under different drilling forces.

Since the experiment required a suitable drilling speed to minimize the operation errors, an appropriate drilling force should be found. To achieve this goal, several experiments were conducted when the rotary speed of the drill was 70 r/min and there was no vibration added on the drill. The results are shown in Figure 14, which indicates that the breaking process of the brick is so weak that the abrasion of the materials takes the main role when the drilling force is weak. When the drilling force is strong, the pretty high drilling speed means that researchers are likely to have considerable reading errors during the recording because the drill will feed downward only about 5 mm. Only in the middle of the figure, the condition is suitable for researchers to perform experiments efficiently and precisely. Finally, 150 N was selected in the experiment.

Based on the preparations above, several core experiments were conducted. In these experiments, the fixed drilling force and the drilling rotary speed were 150 N and 70 r/min. Several conditions were tested in the experiments. These conditions included normal rotary drilling which was applied the excitation voltage of 0 V and vibration assisted drillings which were applied excitation voltages of 100 V, 200 V and 300 V, respectively.

**Figure 15:**
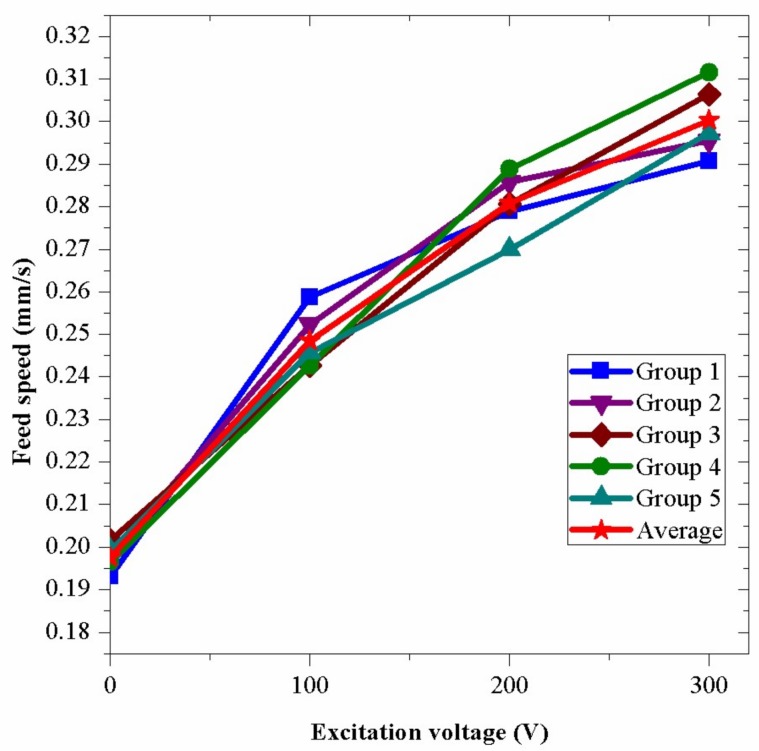
The feed speeds under different excitation voltages.

The results of the drilling feed speed are shown in Figure 15. In the figure, a common characteristic is that, along with the increase of the excitation voltage, the drilling efficiency is improved. In the results, different groups have different feed speeds when the same voltage is applied because the surface conditions of the bricks have some small discrepancies, which results in the differences between the hardness of bricks. In addition, the reading errors contributes to the differences of the feed speeds. In Figure 15, the average feed speed of the vibration assisted drilling is 52% higher than that of the normal rotary drilling when the excitation voltage is 300 V. These results show that the bending vibration increases the feed speed in rotary drilling obviously. The greater the vibration energy is, the more efficient the drilling process is.

**Figure 16:**
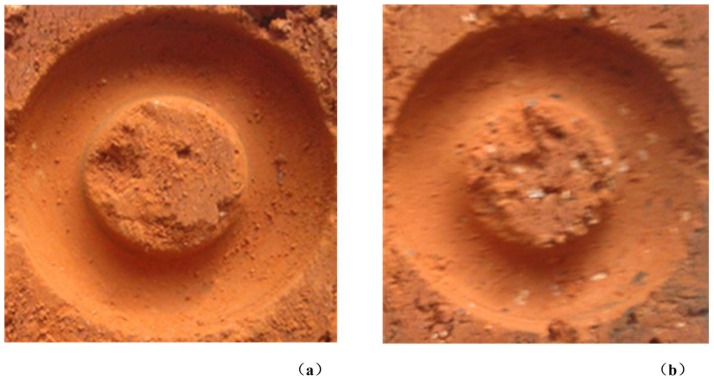
Surface conditions of the drill holes: (**a**) vibration assisted drilling; and (**b**) normal rotary drilling.

Through the observation of the surface of the drill holes, as shown in Figure 16, it is found that the hole of the vibration assisted drilling is smoother than that of the normal rotary drilling. There is also a phenomenon that there are far fewer large rock fragments in the vibration assisted drilling than in the normal rotary drilling, which means that the clearance of the chips is easier in the vibration assisted drilling.

## 6. Conclusions

This paper described the setup of the novel drill assisted with vibrations of frequency of 1269 Hz and tested its efficiency. The proposed drill contained a piezoelectric bending transducer which could generate a circular trajectory vibration on the cut edge of the drill bit by producing two orthogonal bending vibrations. This vibration of the cutting edge helped the drill break the material and feed downward faster. The electric brush is designed to prevent the coiling of the wires as well as conduct electric voltages; the standard cone rod at the tail of the drill is designed to connect the drill with a bench drill which the experiment was based on.

Several experiments are conducted to compare the efficiency of the vibration assisted drilling with that of the normal rotary drilling. The experiments used different excitation voltages (0 V, 100 V, 200 V and 300 V) to change the vibration amplitude of the drill. The results show that the feed speed of the vibration assisted drilling (0.296 mm/s) is about 52% higher than that of the normal rotary drilling (0.195 mm/s) when the excitation voltage is 300 V. The drill holes made by the experiments indicate that the surface condition of the vibration assisted drilling is smoother than that of the normal rotary drilling; furthermore, the rock fragments produced by the vibration assisted drilling are smaller than those of the normal rotary drilling. These results show that the vibration assisted drilling is more efficient than the normal rotary drilling and it can be developed and utilized further in the study of next stage.

## Figures and Tables

**Table 1:** The materials used in the drill.

Part	The Base of Drill Bit	Forepart and Posterior	Electrodes
Material	Carbon steel	Carbon steel	Beryllium bronze
Density (kg/m^3^)	7800	7800	8300
Elasticity Modulus (GPa)	206	206	133
Poisson’s ratio	0.3	0.3	0.35
